# Size of the spatial correlation between ECoG and fMRI activity

**DOI:** 10.1016/j.neuroimage.2021.118459

**Published:** 2021-08-06

**Authors:** Giovanni Piantoni, Dora Hermes, Nick Ramsey, Natalia Petridou

**Affiliations:** a Dept Neurology & Neurosurgery, UMC Utrecht, Heidelberglaan 100, Utrecht 3584 CX, the Netherlands; b Dept Physiology & Biomedical Engineering, Mayo Clinic, Rochester, MN, United States; c Dept Neurology, Mayo Clinic, Rochester, MN, United States; d Dept Radiology, Mayo Clinic, Rochester, MN, United States; e Dept Radiology, UMC Utrecht, Heidelberglaan 100, Utrecht, the Netherlands

**Keywords:** Motor cortex, Electrocorticography, Functional MRI, Functional neuroimaging

## Abstract

Electrocorticography (ECoG) is typically employed to accurately identify the seizure focus as well as the location of brain functions to be spared during surgical resection in participants with drug-resistant epilepsy. Increasingly, this technique has become a powerful tool to map cognitive functions onto brain regions. Cortical mapping is more commonly investigated with functional MRI (fMRI), which measures blood-oxygen level dependent (BOLD) changes induced by neuronal activity. The multimodal integration between typical 3T fMRI activity maps and ECoG measurements can provide unique insight into the spatiotemporal aspects of cognition. However, the optimal integration of fMRI and ECoG requires fundamental insight into the spatial smoothness of the BOLD signal under each electrode.

Here we use ECoG as ground truth for the extent of activity, as each electrode is thought to record from the cortical tissue directly underneath the contact, to estimate the spatial smoothness of the associated BOLD response at 3T fMRI. We compared the high-frequency broadband (HFB) activity recorded with ECoG while participants performed a motor task. Activity maps were obtained with fMRI at 3T for the same task in the same participant prior to surgery. We then correlated HFB power with the fMRI BOLD signal change in the area around each electrode. This latter measure was quantified by applying a 3D Gaussian kernel of varying width (sigma between 1 mm and 20 mm) to the fMRI maps including only gray-matter.

We found that the correlation between HFB and BOLD activity increased sharply up to the point when the kernel width was set to 4 mm, which we defined as the kernel width of maximal spatial specificity. After this point, as the kernel width increased, the highest level of explained variance was reached at a kernel width of 9 mm for most participants. Intriguingly, maximal specificity was also limited to 4 mm for low-frequency bands, such as alpha and beta, but the kernel width with the highest explained variance was less spatially limited than the HFB.

In summary, spatial specificity is limited to a kernel width of 4 mm but explained variance keeps on increasing as you average over more and more voxels containing the relatively noisy BOLD signal. Future multimodal studies should choose the kernel width based on their research goal. For maximal spatial specificity, ECoG electrodes are best compared to 3T fMRI with a kernel width of 4 mm. When optimizing the correlation between modalities, highest explained variance can be obtained at larger kernel widths of 9 mm, at the expense of spatial specificity. Finally, we release the complete pipeline so that researchers can estimate the most appropriate kernel width from their multimodal datasets.

## 1. Introduction

Cortical mapping of cognitive functions relies on multiple techniques that differ in their ability to measure brain activity. One of the most widely employed techniques, functional Magnetic Resonance Imaging (fMRI), relies on the blood oxygenation level dependent (BOLD) signal, whose neuronal correlates are increasingly, yet not fully understood (Hillman, 2014; Logothetis, 2010, 2008). Neuronal activity can be measured directly with electrocorticography (ECoG), which has been widely used in participants with drug-resistant epilepsy to accurately identify the seizure focus as well as the location of brain functions to be spared during subsequent surgical resection (Penfield and Jasper, 1954).

There is an increasing number of studies that combine ECoG and fMRI to investigate brain functions with improved accuracy, since these two modalities exhibit complementary spatial and temporal features (Babajani-Feremi et al., 2018, 2016; Genetti et al., 2015; Jacques et al., 2016; Keller et al., 2013; Lascano et al., 2014; Murta et al., 2017; Sanada et al., 2021; Van Den Boom et al., 2021). This synergy between ECoG and fMRI has translated into direct clinical applications, whereby pre-surgical fMRI and intra-operative ECoG are used to guide the search of epileptogenic zones during resective surgery, with favorable outcomes (Hao et al., 2017; Kamada et al., 2014; van Houdt et al., 2012), also in pediatric patients (Roland et al., 2017).

The comparison of results across modalities requires that they both sample from the same active tissue. The spatial correspondence of the BOLD fMRI and ECoG signal has been previously demonstrated at the millimeter scale at 7T (Siero et al., 2014). However, the optimal integration requires an understanding of the spatial smoothness of the BOLD response. Each voxel under an ECoG electrode has a certain amount of signal and noise. Spatial smoothing may reduce noise, but also lose important signal, whereas a granular response contains more noise and the relative signal may be reduced. Here we study the optimal kernel to relate typical 3T BOLD fMRI with ECoG.

Influential studies have focused on the temporal relationship between the ECoG signal and the sluggish BOLD response (Gaglianese et al., 2017; He et al., 2008; Logothetis et al., 2001; Siero et al., 2013; Sirotin and Das, 2009). Furthermore, a promising line of research has looked at the spectral profile of the neuronal activity in relation to the fMRI signal, identifying the high-frequency broadband (HFB) power changes as the most prominent correlate (Gaglianese et al., 2017; Hermes et al., 2017, 2012; Jacques et al., 2016; Lachaux et al., 2007; Mukamel et al., 2005; Siero et al., 2013).

Subdural electrodes are thought to sample a region of a few millimeters from the contact zone, especially for high-frequency activity (Bédard et al., 2006; Buzsáki et al., 2012; Dubey and Ray, 2019), although the exact extent likely depends on the size and shape of the electrodes and on the underlying tissue (e.g. if located in the proximity of a gyrus or a sulcus). On the other hand, the spatial resolution of fMRI is related to the hemodynamic properties, acquisition parameters, voxel size, and vasculature of the sampled brain region.

A common approach in comparing ECoG and fMRI measures is to define a spherical mask around each electrode, which is then used to extract the region of interest (ROI) from fMRI data. The radius of the sphere remains a free parameter. This measure is variably taken to be 5 mm (Jacques et al., 2016), 8 mm (Gaglianese et al., 2017; Hermes et al., 2012) or 10 mm (Kubanek and Schalk, 2015). However, a pure spheric model is a simplified approximation of the underlying physiological process, as neuronal populations closer to the electrode location have a larger effect on the recorded signal than distant locations (Einevoll et al., 2013).

Here, we explicitly model this bias for proximal regions, by implementing a 3-dimensional Gaussian kernel model, where the only free parameter is the width of the kernel (reported as sigma, which was identical in the 3 dimensions). We set up to systematically find the most appropriate sigma value for the Gaussian kernel, by measuring the spatial correlation between ECoG and fMRI measurements of the same motor task, in the same cohort of participants. An empirically derived estimate of this measure would be highly beneficial to not only cross-modal research but also clinical mapping (van Houdt et al., 2012), by providing a more accurate co-localization of the neuronal generators observed with ECoG and fMRI. This improved localization would have a wide range of applications: from guiding surgeons in planning resective surgery to selecting the optimal placement of ECoG electrodes for brain-computer interface (BCI) applications on the basis of fMRI results (Van Den Boom et al., 2021).

To this aim, we assumed that motor activity under the ECoG grids display regional selectivity on the surface of the brain, rendering a range of values across electrodes, associated with a varying degree of presence or absence of activity associated with the activity pattern, in underlying tissue. This variance in activity under electrodes was used to determine the maximum agreement between the HFB measure in ECoG and BOLD fMRI across a range of smoothing kernels for the fMRI data. Of note, this analysis does not account for the sulcal boundaries within sensorimotor regions, or between sensorimotor and other regions, but it does allow for estimation of a generic point-spread function for BOLD fMRI in relation to spatially confined ECoG HFB measurements.

## 2. Materials and methods

### 2.1. Participants

Participants were 20 patients (10 females) with drug-resistant epilepsy and candidate for resective surgery, who were admitted for intracranial epilepsy monitoring at the University Medical Center Utrecht. Average age of the patients at the time of implantation was 21.5 years (s.d. 11.31, range: 8–49) and 11 patients were under the age of 18. Before the fMRI session written informed consent to participate in this study was given by all the patients (and their parents / legal guardians for pediatric patients). The study was approved by the ethical committee of the University Medical Center Utrecht, in accordance with the Declaration of Helsinki (2013).

### 2.2. Task

During a preoperative fMRI session and during ECoG recordings, participants performed a block-design motor task with 30 s periods of rest interleaved with 30 second periods of finger movement, for 4.5–5.5 min in total, as described previously (Hermes et al., 2012). During the finger movement period, patients were instructed to either flex and extend the thumb or flex and extend all the fingers of the hand which was contralateral to the implantation site. Patients performed the same movement during both the fMRI and the ECoG session. Patients were cued to flex and extend their fingers when a green circle appeared on the screen (green circle was on the screen for 250 ms, followed by a fixation cross for 250 ms), so that the movement followed a rhythm of approximately two movements per second. The stimulus for the rest period was identical to the movement period, with the exception that the circle was red. The total duration of the task in the MRI scanner was 4 min and 30 s (4 movement periods and 5 rest periods) and the total duration of the task during the ECoG recording was 5 min and 30 s (5 movement periods and 5 rest periods).

### 2.3. fMRI data acquisition

fMRI data were acquired on a Philips Achieva 3 T scanner using 3D PRESTO (Neggers et al., 2008; van Gelderen et al., 2012) and a 8-channel head coil. 40 slices were acquired with a field of view (FOV) of 224 × 256 × 160 mm^3^ and with a voxel size of 4 mm isotropic. Volume-to-volume repetition time (TR) was 0.608 s, with a flip angle of 10°, echo time (TE) of 33.2 ms, and TR between subsequent RF pulses of 22.5 ms. In addition, whole-brain T1-weighted 3D TFE structural images were acquired at a resolution of 1 mm isotropic, with FOV: 288 × 288 × 175 mm^3^; flip angle: 8°; TR: 8.4 ms; TE: 3.8 ms.

### 2.4. MRI / fMRI preprocessing

In Freesurfer (Fischl, 2012), individual T1-weighted images were segmented to generate a gray matter mask and smooth pial surfaces of the brain. fMRI data processing was carried out using FEAT 6.00, part of FSL (Jenkinson et al., 2012). Registration to the high-resolution structural images was carried out using FLIRT (Jenkinson et al., 2002). The following pre-processing was applied: motion correction using MCFLIRT (Jenkinson et al., 2002); skull removal using BET (Smith, 2002); grand-mean intensity normalization of the entire 4D dataset. High-pass temporal filtering was achieved with a Gaussian-weighted least-squares straight line fitting (sigma = 45 s). One patient had a volume-to-volume mean displacement of 0.303 mm and was therefore excluded from the subsequent analysis. For all the remaining participants included in the study, volume-to-volume mean displacement was on average 0.119 mm (range: 0.025–0.167 mm) and overall mean displacement was on average 0.627 mm (range: 0.164–1.162 mm) across participants.

**Fig. 1.**
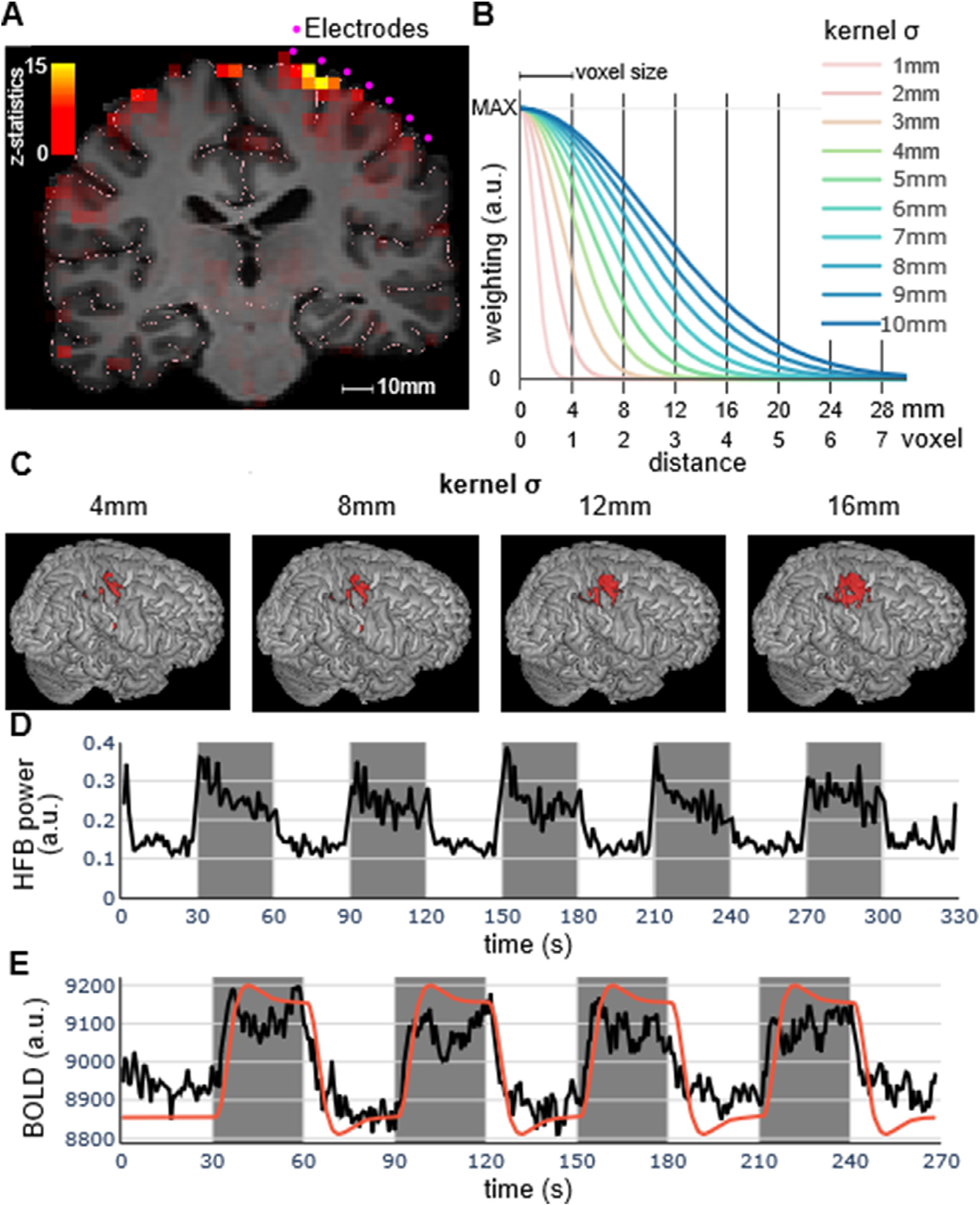
(A) Z-scores for a motor task at 3T fMRI, alternating 30 s blocks of movements and 30 s blocks of rest. Results shown here are superimposed on the structural MRI. Only voxels containing gray matter were included in the analysis. Each electrode (indicated in purple) was localized on the pial surface (delineated in pink). (B) We computed the weighted average of the z-scores of the voxels surrounding the each electrode. The weights were based on a 3D Gaussian kernel of varying width (for illustration purposes, we show here kernels with sigma between 1 and 10 mm). (C) Illustration of the degree of 3D Gaussian smoothing for different kernel width (4 mm corresponds to 1 voxel). (D) Time course for ECoG activity for the electrodes with a z-score > 10 for one participant (*P01*). Dark background indicates the 30 s movement period and light background indicates the 30 s rest period. (E) Time courses for BOLD activity for the voxels with a z-score > 10. As above, dark and light background indicates movement and rest periods, respectively. The red line indicates the regressor convolved with a standard HRF (in arbitrary units).

Statistical analyses were performed on a single-subject basis in native space and therefore no smoothing was applied. A GLM was estimated with one regressor for hand movement activation, i.e. a 30s box car for movement blocks convolved with a standard hemodynamic response function (HRF), and was used to generate whole-brain activity maps with z-scores for each voxel. Only voxels containing gray matter were included in the subsequent analysis (Fig. 1A).

### 2.5. ECoG data acquisition

Grid electrodes (AdTech, Racine, WI) had a measurement surface of 2.3 mm diameter, with 10 mm inter-electrode spacing, and were positioned directly on the cortical surface. The placements of the electrodes was based on purely clinical reasons (i.e. to identify the epileptogenic zone) and differed across patients (Supplementary Figs. 1–3). One participant (*P11*) had, in addition to electrodes spaced at 10 mm, an 8 × 4 grid of electrodes with the same diameter and material, whose inter-electrode spacing was 5 mm. A reference electrode was positioned extra-cranially on the mastoid bone. Recordings were acquired on a 128-channel Micromed system (Treviso, Italy), with a sampling rate of 512 Hz and band-pass filtered between 0.15 and 134.4 Hz. Electrodes were localized from an MRI-coregistered post-implantation computed tomography (CT) scan of the head. To correct for the brain shift, electrodes were projected onto the pial surfaces in the direction of the norm of the grid (Hermes et al., 2010)

### 2.6. ECoG preprocessing

Channels exhibiting low signal or epileptic artifacts were rejected, as assessed by an automatic procedure excluding channels that exhibited a variance larger or smaller than 3 standard deviations of the variance of other channels (Liu et al., 2015). Signals from remaining electrodes were referenced to the common average. Data was separated for the active and rest conditions. For each condition separately, the power spectrum was then computed on 2 s long windows after applying a Hanning taper. We calculated the HFB activity by taking the average across 0.5 Hz frequency bins of the log-transformed power spectrum density in the 65–95 Hz frequency range for each time window (Hermes et al., 2012). For each electrode, we compared the HFB activity during the active period against the rest period, by calculating an unpaired two-sample *t*-test over the 2 s long windows. Because we expected that the BOLD activity only reflects task-related increase (and decrease) in neuronal activity, we excluded electrodes that did not show a significant difference between the two conditions (*p*-value was set at 0.05) from the subsequent analysis. Because this step was only necessary to exclude electrodes that did not show a change in neuronal activity and was not the main outcome of this study, we did not correct for multiple comparisons.

Due to their involvement in motor activity (Hummel et al., 2002; Pfurtscheller et al., 1996; Ramos-Murguialday and Birbaumer, 2015), the same procedure was then performed on the alpha frequency band (8–12 Hz) and beta frequency band (13–30 Hz). The results for these frequency bands are presented separately.

### 2.7. Peak correlation between ECoG and fMRI

The procedure described in *ECoG Preprocessing* defines z-scores for each electrode representing the difference in HFB activity between movement and rest. These values were compared with the z-scores computed from the fMRI BOLD signal change in the area around each electrode. The correlation was computed for each participant to compensate for the systematic differences in activation strength within individuals, and to account for the varying electrode locations across individuals. The fMRI z-score computed at each electrode was the weighted average of the fMRI z-scores for the gray-matter voxels surrounding that electrode. The weights were based on the distance between the center location of each electrode and the center of the fMRI voxels, multiplied by a Gaussian kernel of varying width. This procedure was repeated for a range of kernel widths (sigma between 1 mm and 20 mm, Fig. 1B). In this way, we obtained a weighted average fMRI z-score at each electrode, for each kernel width (Fig. 1C). Finally, we correlated the ECoG z-scores across electrodes with the corresponding fMRI z-scores, as a function of the width of the 3D Gaussian kernel used to compute the fMRI z-scores. We then identified the kernel width at which the correlation between the ECoG z-scores and the fMRI z-scores was the highest (in terms of explained variance, *r*^*2*^).

### 2.8. Concavity

With increasing kernel width, the BOLD fMRI measure becomes more stable as noise is averaged out across voxels, which results in higher correlation with ECoG. Yet, with increasing kernel width (i.e. increased smoothing of the fMRI signal), the pattern of brain activity is increasingly unspecific. The kernel width at which the pattern is lost, is dependent on the spatial features of the pattern itself, including size of an active region, proximity to other active regions, and distribution of the activation over the cortex. This pattern of activation is assumed to vary across participants sufficiently to constrain a particular bias in the correlation measures. Therefore, two factors play a role in assessing the spatial correlations between ECoG and fMRI measures across kernel widths: 1) the point-spread function of BOLD response for a single source of brain activity (the variable of interest of this study), 2) the decline in fMRI noise with increased spatial averaging. These cannot be disentangled well, so we adopted two measures of optimal ECoG-fMRI fit: 1) the peak correlation across kernel widths, 2) the point at which the gain in correlation with increasing kernel widths tapers off.

Therefore, in addition to identifying the kernel width at which the correlation was the strongest (“peak correlation”), we determined the kernel width after which the improvement in correlation strength was only marginal (“concavity”). This problem can be solved by finding the kernel width at which the downwards concavity of the kernel-width/r^2^ curve was the highest. Mathematically, this approach corresponds to taking the second derivative of the curve and identifying the lowest trough.

### 2.9. Code availability

Data were organized according to the BIDS format (Gorgolewski et al., 2016; Holdgraf et al., 2019). The complete analysis pipeline is available online (https://github.com/umcu-ribs/grvx) and can be applied to any BIDS-compatible multimodal dataset containing ECoG and fMRI data.

## 3. Results

### 3.1. ECoG results

Twenty participants with intracranial recordings performed a simple hand motor task in which they were asked to alternatively move their fingers for 30 s and to relax the hand for 30 s. One participant was excluded for excessive head motion during the fMRI session. The time course of one ECoG session is represented in Fig. 1D). On average, we included 78.895 electrodes (standard deviation 26.565, range [47–120]) per participant. The highest z-score was, on average, 17.056 (s.d. 7.918 range [4.677–35.021]) and the lowest z-score was −4.661 (s.d. 1.376, range [−8.650 to −2.566]). In total, 40.37% (s.d. 12.77, range [19.61–63.83]) of the electrodes showed significant signal changes during the motor periods as compared to the baseline periods. Results for individual participants are reported in Table 1.

For the alpha frequency band, the highest z-score was, on average, 3.563 (s.d. 1.970, range [0.012–6.576]) and the lowest z-score was −12.838 (s.d. 7.078, range [−28.353 to −0.603]). In total, 51.40% (s.d. 25.25, range [20.00–94.55]) of the electrodes showed significant signal changes during the motor periods as compared to the baseline periods. For the beta frequency band, the highest z-score was, on average, 4.656 (s.d. 3.368, range [0.380–12.910]) and the lowest z-score was −13.497 (s.d. 5.790, range [−24.584 to −2.096]). In total, 51.92% (s.d. 20.54, range [27.66–88.52]) of the electrodes showed significant signal changes during the motor periods as compared to the baseline periods.

### 3.2. fMRI results

The same cohort of participants performed the same task in a 3T MRI scanner before the ECoG recording period. The time course of one participant is represented in Fig. 1E). The fMRI analysis revealed that the highest voxel z-score averaged across participants was 14.167 (s.d. 4.052, range [7.116–21.848]) and the lowest voxel z-score was −8.963 (s.d. 2.458, range [−12.328 to −3.338]). In total, 12.20% (s.d. 8.66, range [2.07–29.80]) of the voxels showed significant signal changes during the motor periods as compared to the baseline periods, after correcting for multiple comparisons at the cluster level (Woolrich et al., 2004). Results for individual participants are reported in Table 2.

### 3.3. Peak correlation between ECoG and fMRI

**Fig. 2.**
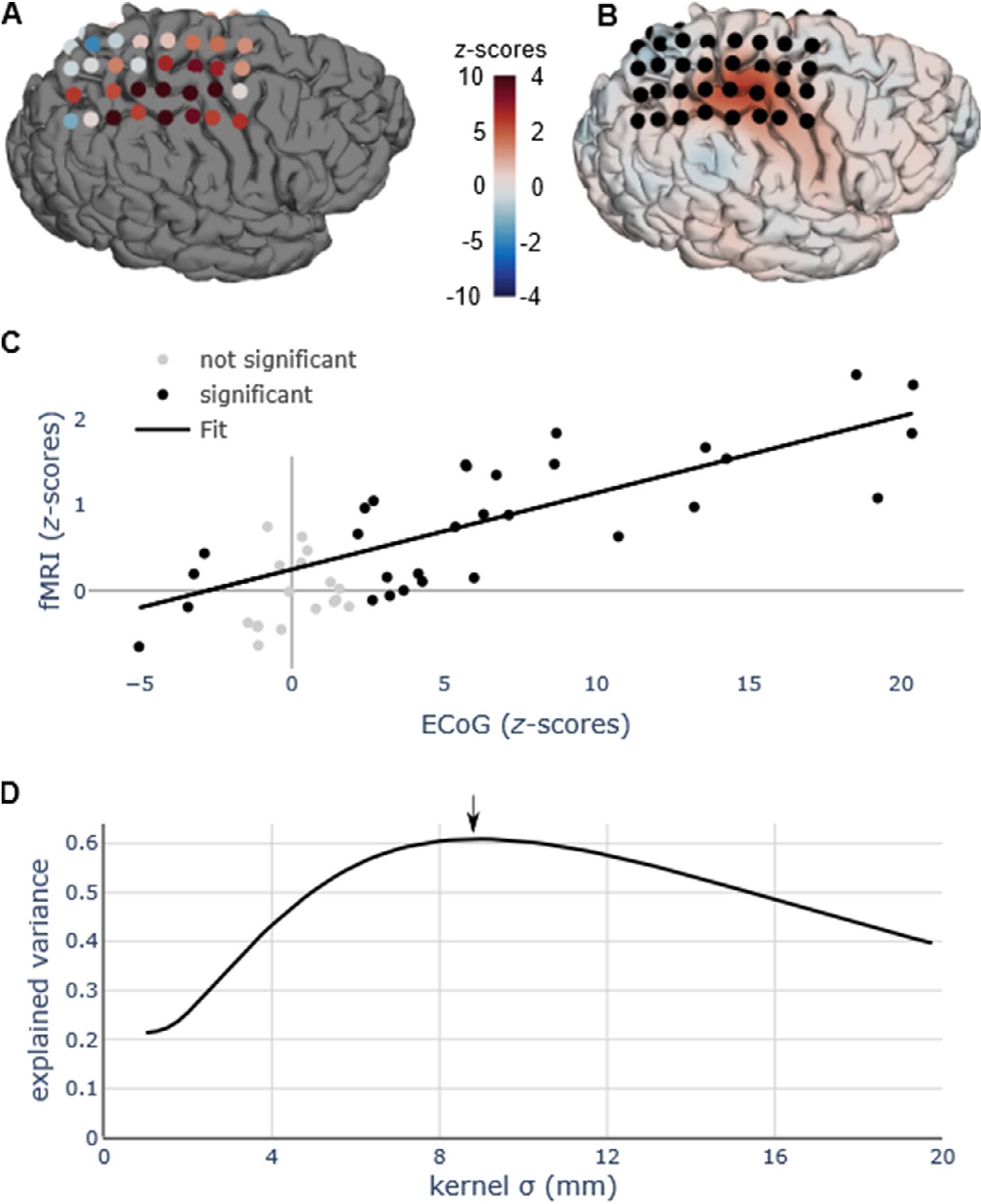
(A) Spatial distribution of the z-scores for all the electrodes for one participant (*P01*). (B) Projection to the surface of the z-scores during the fMRI session for the same participant, smoothed with a 3D kernel width of 9 mm (as it was found to be the optimal kernel width). (C) Correlation between ECoG z-scores and fMRI z-scores for one kernel width, which, in this case is the kernel width with the strongest correlation (9 mm), as marked by an arrow in panel (B). Only electrodes showing a significant ECoG activation (both positive or negative) were used to compute the correlation (black circles), while electrodes without a significant ECoG activation are shown in gray and were not used for the computation of the ECoG-fMRI correlation. (D) Correlation, measured as explained variance (*r*^2^), between fMRI and ECoG z-scores, as a function of the width of the Gaussian kernel. For this participant, *r*^2^ peaks at around 9 mm and the correlation corresponding to this peak is shown in panel (A). Results shown in panel A, B, and D for all the participants are shown in Supplementary Figs. 1–3.

We correlated the changes in ECoG signal during a motor task with the changes in fMRI signal during the same motor task (z-scores for one participant for ECoG and fMRI session are shown in Fig. 2A and B, those for all the participants in Supplementary Figs. 1–3). We observed a positive correlation between HFB activity and BOLD activity across electrodes (results for one participant are shown in Fig. 2C). Explained variance (*r*^*2*^) was computed at multiple widths of the 3D Gaussian kernel, that was used to compute the weighted average of the fMRI signal change around each electrode. We observed that explained variance was low for small (< 4 mm) kernel widths and increased until 9 mm (exemplary results from one participant are shown in Fig. 2D, results for all participants in Supplementary Figs. 1–3). After that point, increasing the kernel width either hardly improved or even decreased the explained variance.

**Fig. 3.**
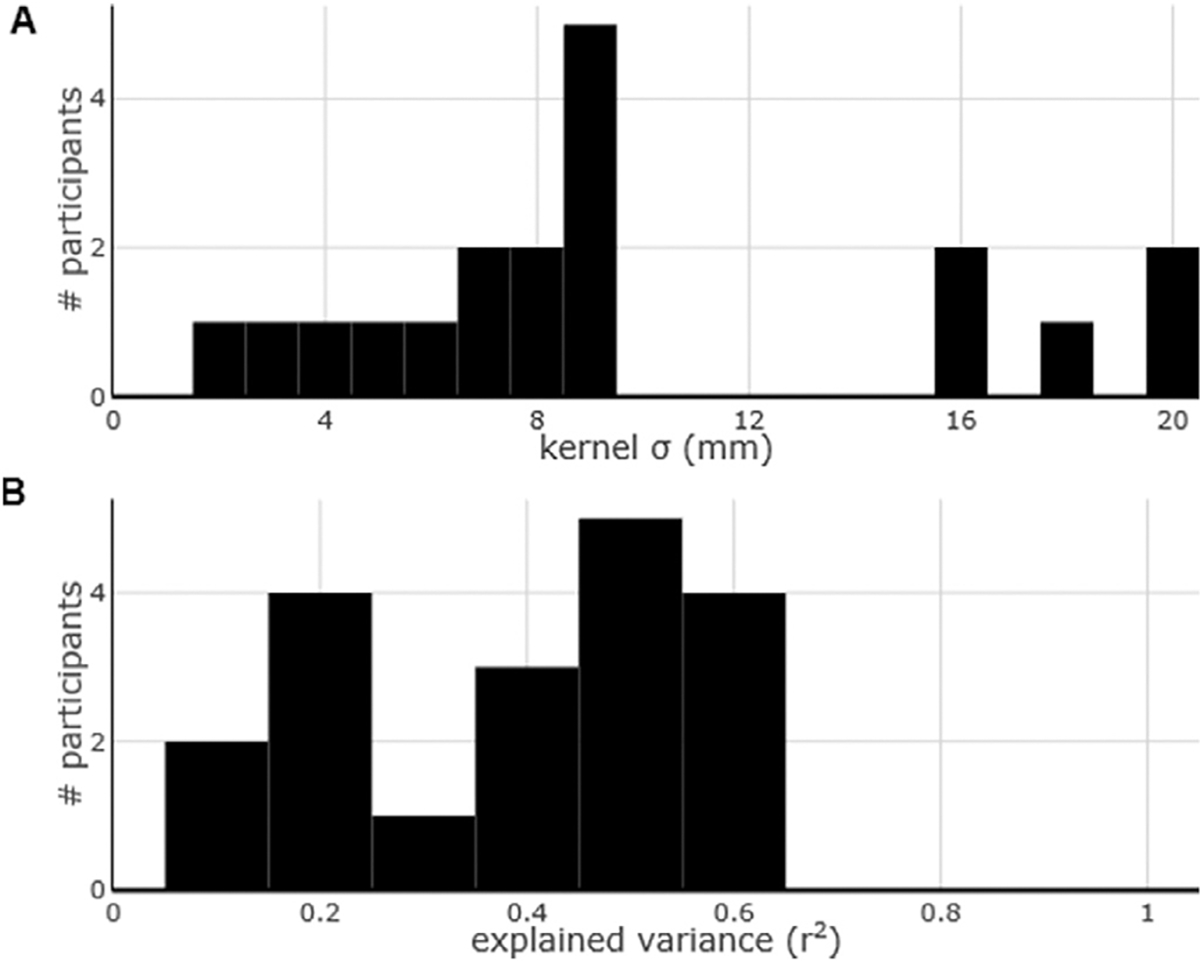
Group results of the best kernel width and maximum explained variance. (A) The kernel width that explained the most variance across all the participants was at around 9 mm. (B) Maximum explained variance of the fMRI measurements based on the ECoG measurements across all the tasks for all the participants.

This relationship was consistent across most participants as the maximum explained variance was observed at kernel widths between 7 and 9 mm for nine participants, with a mean of 9.566 mm (s.d. 5.422) (Fig. 3A). All the correlations were positive (average BOLD / ECoG slope: 0.135, s.d. 0.101), indicating that larger ECoG signal change corresponds to larger fMRI signal change. The average degree of explained variance was 39.06% (s.d. 16.42, range: 10.08–60.92%, Fig. 3B).

### 3.4. Concavity

**Fig. 4.**
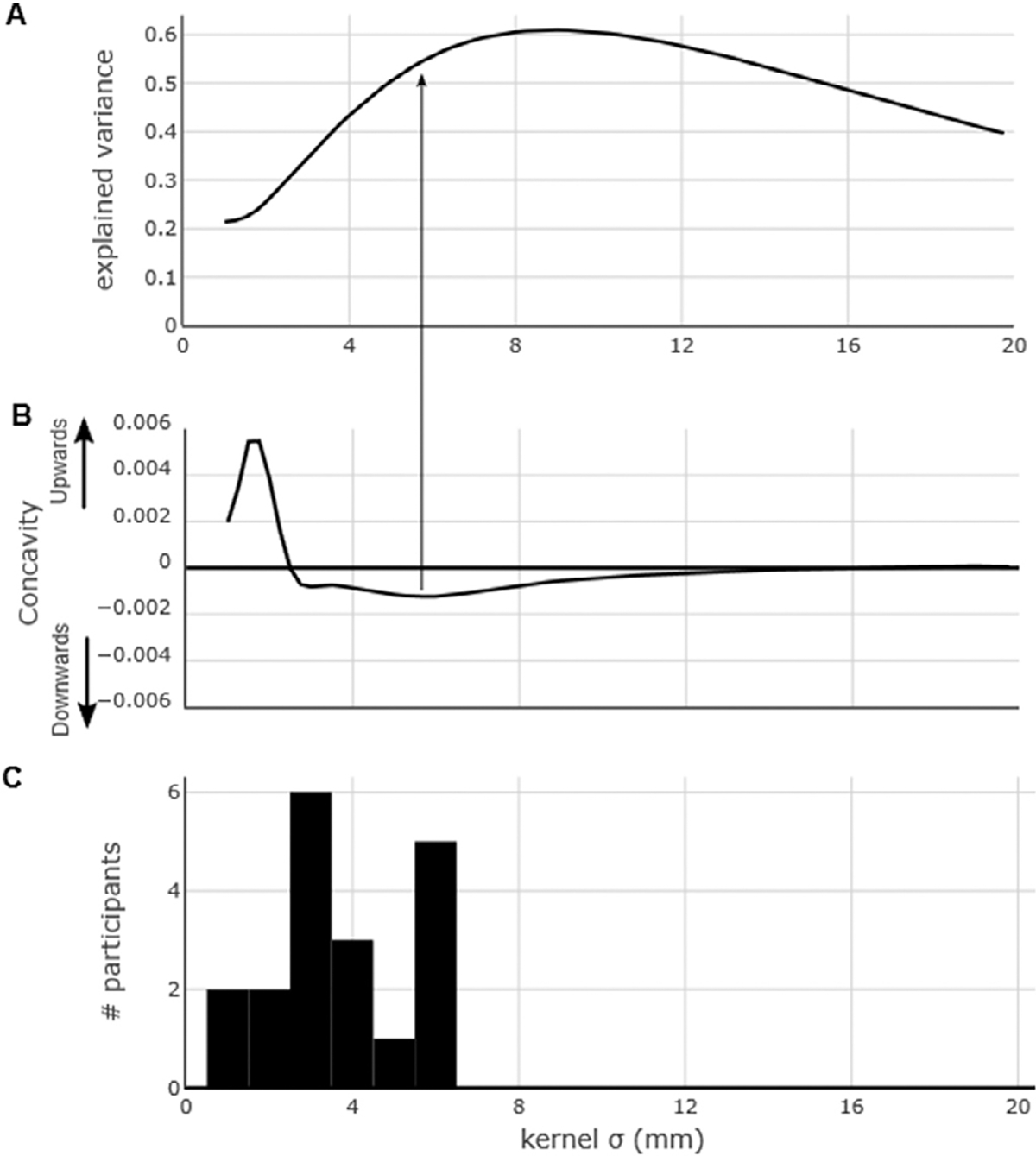
(A) As Fig. 2B, for reference. (B) Second derivative of the curve in (A). The most negative value of the second derivative represents the point of maximum downwards concavity, i.e. the point after which increasing kernel width improves the level of explained variance only marginally. (C) Histogram of the points of maximum downwards concavity across all participants.

Finally, we noticed that the level of explained variance increases markedly at the lowest kernel widths but tapers off (or even decreases) as the kernel width is larger than a few millimeters. To quantify the point at which the increase of explained variance starts diminishing, we computed the second derivative of the explained-variance / kernel-width line (Fig 4B). We observed, across participants, that this inflection point occurred on average at 3.697 mm (s.d. 1.574) and was ≤ 6.25 mm for all participants (Fig 4C). The average degree of explained variance at the point of maximum downwards concavity was 29.54% (s.d. 12.75%).

### 3.5. Alpha and beta frequency bands

**Fig. 5.**
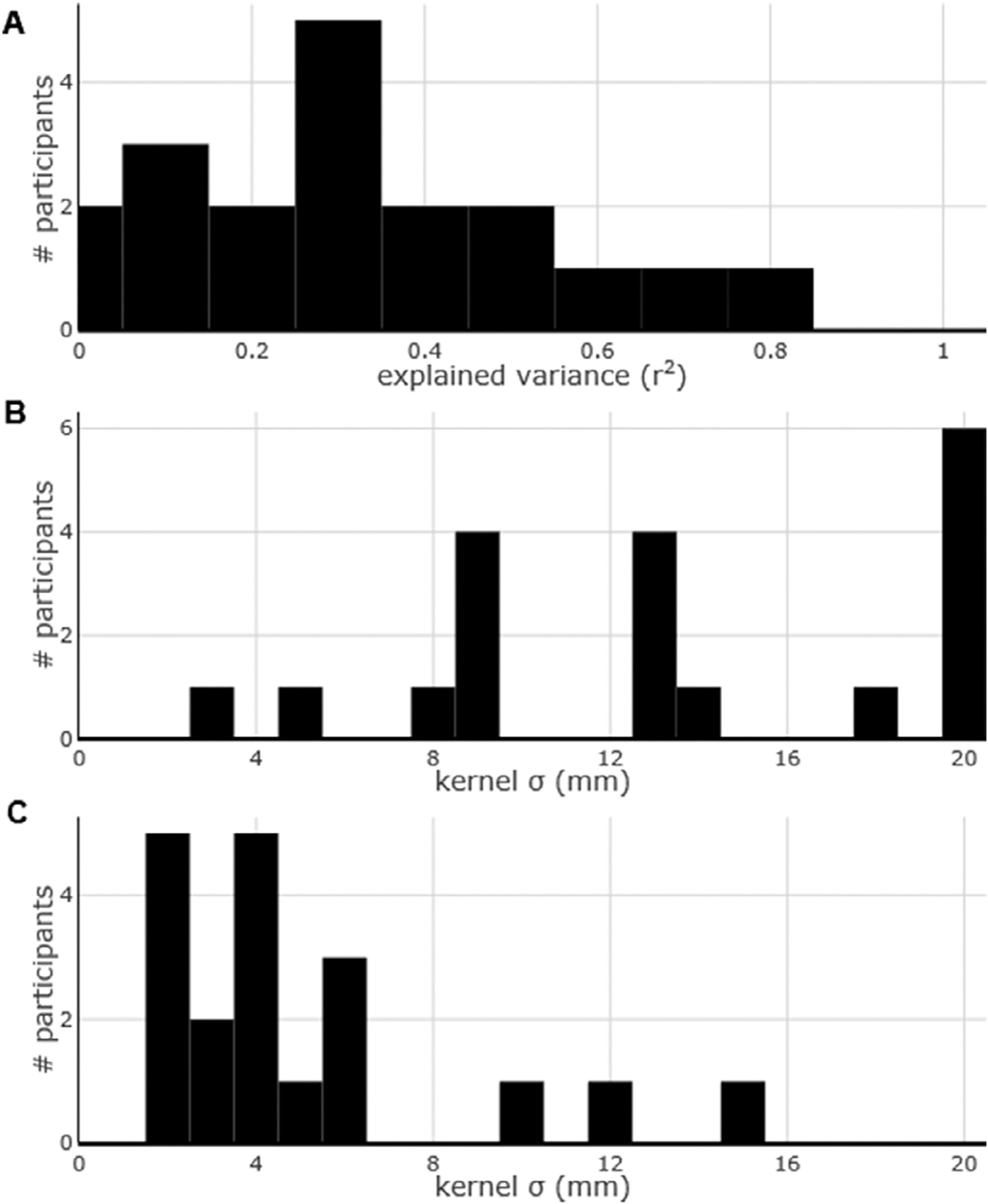
Group results for the alpha frequency band (8–12 Hz). (A) Maximum explained variance of the fMRI measurements based on the ECoG measurements across all the tasks for all the participants. (B) The optimal kernel width across all participants. (C) Histogram of the points of maximum downwards concavity across all participants.

**Fig. 6.**
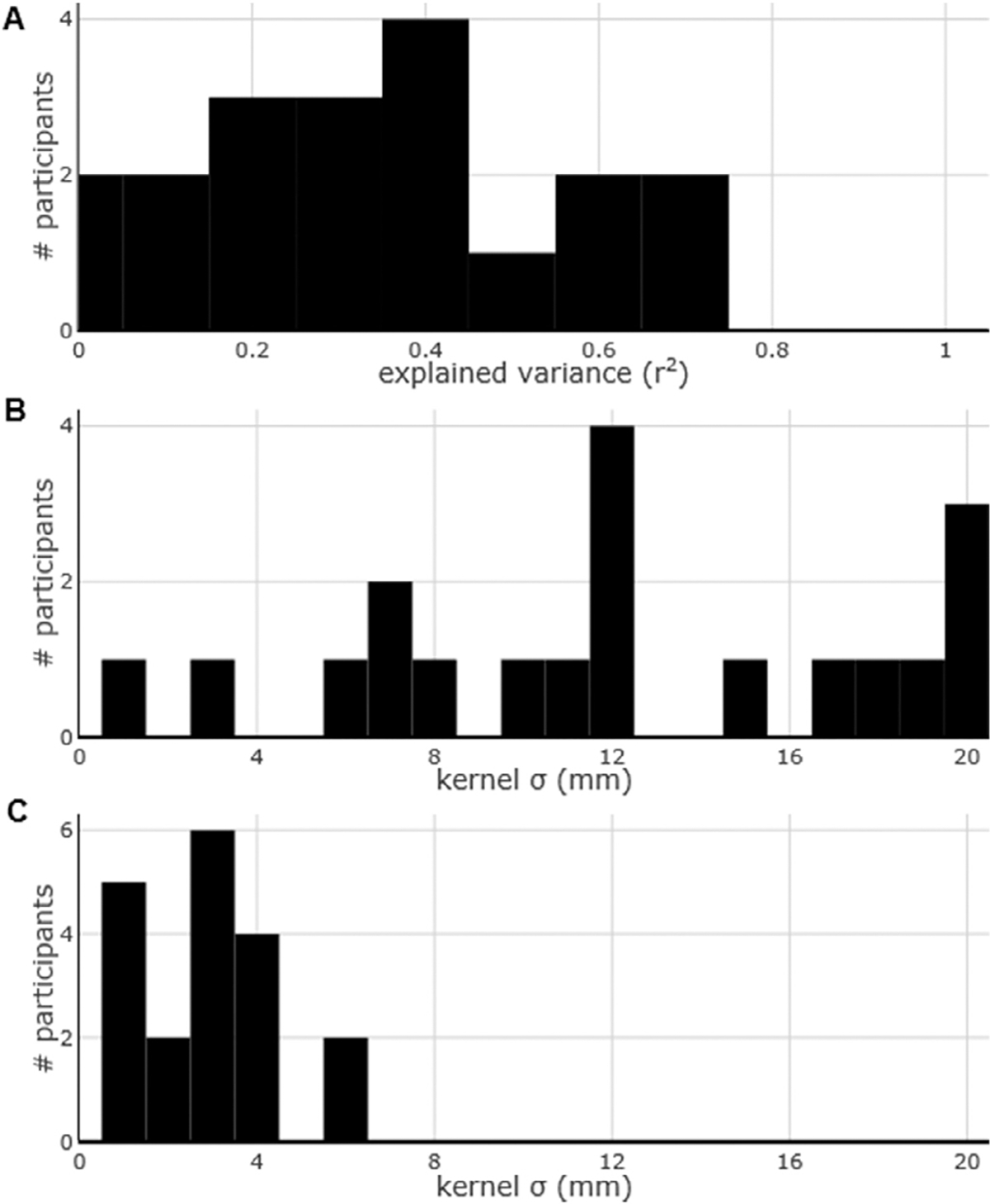
Group results for the beta frequency band (13–30 Hz). (A) Maximum explained variance of the fMRI measurements based on the ECoG measurements across all the tasks for all the participants. (B) The optimal kernel width across all participants. (C) Histogram of the points of maximum downwards concavity across all participants.

We performed the same analysis above on the alpha (8–12 Hz) and beta (13–30 Hz) frequency bands. The results are reported in Fig. 5 for alpha and in Fig. 6 for beta. We found that the correlation between the activity in both these frequency bands and the BOLD activity was generally very high across participants (Figs. 5A and 6A). In fact, the explained variance was as high, if not in some cases higher, that the explained variance between HFB and BOLD activity. However, the distribution of the kernel width with highest explained variance was much more scattered for the alpha frequency band, in some cases not reaching a maximum even at the threshold of 20 mm (Fig. 5B). The kernel width with highest explained variance for the beta frequency band was also rather scattered, but for some participants it lay at around 12 mm (Fig. 6B). Intriguingly, the distribution of the points of maximum downwards concavity was, for alpha and beta, within 4 mm for almost all the participants (Figs. 5C and 6C), similarly to the HFB activity (Fig. 4C).

A major difference between the HFB activity and the low-frequency bands is the sign of the slope of the correlation between ECoG and BOLD activity. For the HFB, higher ECoG activity correlated with higher BOLD activity while the opposite was true for alpha (BOLD / ECoG slope: −0.051, s.d. 0.224) and beta (BOLD / ECoG slope: −0.049, s.d. 0.154).

## 4. Discussion

This study investigates the spatial relationship between 3T BOLD fMRI and electrophysiological changes in high frequencies with ECoG. Brain activity was measured with both modalities in a cohort of 19 participants who performed the same simple motor task, which generates a consistent and reliable pattern of neuronal response (Ramsey et al., 1996). We then computed the spatial correlation between the direct and indirect measures by systematically varying the width of the Gaussian kernel used to average the BOLD activity around each electrode. We found that the maximum correlation between HFB signal change in the ECoG electrodes and fMRI BOLD signal change was observed with a kernel width of 9 mm of the latter on average across participants. In line with earlier reports (Gaglianese et al., 2017; Hermes et al., 2012; Jacques et al., 2016; Siero et al., 2013), we observed that the relationship between HFB activity was correlated with the BOLD activity measured around the corresponding electrodes (results from one participant at one kernel width, Fig. 2A).

We speculate that the kernel width with the highest multimodal correlation identified in our study is affected primarily by three intertwined factors. The first factor is the point-spread function which links the activation of a single patch of cortex to the corresponding fMRI activation area (Fracasso et al., 2021). This point-spread function is influenced by a complex mechanism which generates a BOLD response from the firing of a population of neurons (Hillman, 2014; Logothetis, 2010, 2008), on top of the effect of draining blood vessels onto the BOLD signal (Turner, 2002). The second factor is the underlying spatial pattern of the brain response. A highly localized focus of activation as compared to distributed generators would give a smaller kernel width. The third factor is of statistical nature: wider kernel widths average over a larger number of voxels, making its estimate more robust. Robust estimates of the fMRI response in turn result in higher correlation with the ECoG values, which creates a bias towards wider kernel widths.

Disentangling the contribution of each factor is not possible without systematically manipulating the experimental design to account for all these variables, which was not feasible under our experimental paradigm. However, we can lessen the influence of the third factor by focusing on the kernel width at which the improvement in correlation strength was only marginal. This approach, which consists in taking the point of maximum downwards concavity (second derivative), indicates that the kernel width after which the increase in variance starts diminishing is on average around 3–4 mm (Fig. 4), giving a measure of the degree of spatial specificity. This result is in line with the findings of (Siero et al., 2014), where 7T BOLD and HFB activation foci were co-localized within 3 mm. The apparent consistency of this measure across participants and frequency bands suggests that this kernel width represents an intrinsic property of the neurovascular response, the generic point-spread function, instead of being the result of the underlying spatial activation. The spatial activation was in fact variable across participants, due to the variability in electrode locations and in brain response (Supplementary Figs. 1–3). Therefore, we suggest that our approach to maximize spatial specificity is able to capture the generic point-spread function, which links the highly local electrophysiological activation to the BOLD fMRI activation maps. It should be noted that the BOLD point-spread function varies across cortical depth according to the distribution of the draining vessels, as suggested by laminar fMRI studies at ultra-high field (Fracasso et al., 2021; Koopmans et al., 2010; Polimeni et al., 2010).

Low-frequency activity, especially decrease in beta frequency band, is strongly involved in motor tasks (Crone et al., 1998; Pfurtscheller et al., 1996). In contrast to HFB activity, which is known to be highly localized (Dubey and Ray, 2019), low-frequency activity is thought to involve widespread cortical areas (Crone et al., 1998; Lindén et al., 2011; Miller et al., 2007). Our findings did not identify a clear peak in the preferred kernel width between alpha and BOLD activity during the motor task, in line with the observation that anatomical consistency was lower of low-frequency bands (Hermes et al., 2012; Kucyi et al., 2018). On the other hand, the degree of explained variance was high for both high-frequency and low-frequency activity. The disassociation, across frequency bands, between the consistently high explained variance and the heterogeneous distribution of the variable optimal kernel widths may be explained by the observation that low-frequency activity explain part of the BOLD signal which is not explained by the HFB activity (Hermes et al., 2017).

### 4.1. Interpretation of the spatial neurovascular response

Electrodes are thought to record from the cortical area directly underneath the contact (Dubey and Ray, 2019), and therefore we take the neuronal response measured with ECoG as the ground truth to estimate the spatial resolution of the BOLD response. This discrete focus of neural activity translates to a vascular response with a complex vascular architecture. Because of the vascular origin of the BOLD signal, the activation region is effectively blurred by the presence of draining vessels (Turner, 2002). For these reasons, even if no additional smoothing was applied in the fMRI preprocessing pipeline, the generic point-spread function can effectively be thought of as a smoothing filter of the spatial activity (Fracasso et al., 2021). Here we used PRESTO fMRI acquisition, a technique that suppresses intra-vascular signals from larger vessels (Neggers et al., 2008; van Gelderen et al., 2012), however we cannot fully exclude that our signal is sensitive to the extravascular component. For EPI acquisitions, the BOLD signal would in principle include both intra- and extra-vascular signals at 3T. Future approaches to assess the exact contribution of larger vessels could apply multiple fMRI sequences that selectively suppress or enhance the contribution of various vascular compartments.

### 4.2. Limitations

Part of the unexplained variance can be due to a series of factors that could be only partially mitigated. First, the data acquisition necessarily took place on different days, where the state of the participant (e.g. concentration, or tiredness) may differ. However, test-retest studies with fMRI have shown a highly consistent spatial pattern of activation across days (Friedman et al., 2008). In this study, we included a relatively large number of participants, which should average out the day-to-day variability and return a robust estimate of the spatial correlation for this task.

Second, the degree of correlation might be affected by the spatial configuration of the electrodes on the cortex, in two ways. Electrodes on the clinical grids are spaced 10 mm apart and cover only regions of interest for epilepsy monitoring. Given that these grids in effect only sample from 4% of the cortical surface covered by the silicon sheet (at 1 electrode per 100 mm^2^ with an electrode surface of 4.2 mm^2^), it is possible that some of the active regions as measured by whole-brain fMRI are missed. We were able to identify significant electrodes over the motor cortex in all participants, indicating that the HFB recordings in our study captured task-related brain activity, albeit possibly not the epicenters of activity. In addition, localization errors may have occurred in estimating the location of the grid electrodes on the cortex. Several steps were taken for determination of the electrode locations (MR-CT coregistration, determination of the electrode center of mass, brain shift correction), which likely left some inaccuracy. Previous studies, including work from our group, have shown, however, that this localization error is less than 2 mm, in comparison to pictures taken during both the implantation and explanation surgery (Branco et al., 2018; Dykstra et al., 2012; Hermes et al., 2010).

Third, this analysis is not limited to the primary sensorimotor cortex, but includes all the electrodes that were significant during the execution of the task. By including all the areas around electrodes that were significantly activated by the task, we were able to estimate a generic point-spread function for BOLD fMRI in relation to spatially confined ECoG measurements, which was not limited to the sensorimotor cortex. However, even if we included multiple brain areas in the analysis, we did not specifically account for activation patterns that had multiple foci. This effect could confound our findings, as proximal distributed neural generators could result in wider kernel width estimates. Looking at the individual activation maps for ECoG and fMRI (Supplementary Figs. 1–3), there does not seem to be a relationship between the spatial distribution of the brain activity measured with each technique and the kernel width with highest explained variance. The comparison between *P14* and *P15* exemplifies this point: both patients had a highly focal HFB activation and a diffuse BOLD activation (Supplementary Fig. 3). Yet, *P14* shows a clear preference for a kernel width at 7 mm, while the kernel width curve for *P15* is in fact reversed.

We propose that this variability across participants is best controlled for by employing the maximum downwards concavity analysis, which indicates the spatially specific extent of the correlation between ECoG and fMRI activity. By using this approach, we see that the kernel width with the highest spatial specificity is limited to a radius of 4 mm, which roughly corresponds to the voxel size in the current study (Fig. 4C).

### 4.3. Implications for future studies

In light of the previous considerations, we propose that the optimal kernel width for future multimodal studies depends on the research aims. The maximal spatial specificity between 3T fMRI and ECoG measurements can be obtained by using a 3D Gaussian kernel of 4 mm width. This relationship holds true across low (alpha and beta bands) and high (HFB) frequency ranges. Highest correlation between modalities can be obtained by increasing the kernel width, at the cost of poorer anatomical specificity. This increase in explained variance is likely due to the increase in the number of voxels used to compute the average of the relatively noisy BOLD signal.

It remains an open question whether these results regarding the spatial extent of the neurovascular response generalize to more refined measures of brain activity, such as phase-amplitude coupling (PAC) (Murta et al., 2017) or to other cognitive or resting-state tasks. Considering that the variability in the spatial extent of the ECoG activity across frequency bands did not affect the concavity metric (compare Figs. 4C–6C), we expect that the kernel width at which maximal specificity is observed will hold for a variety of brain-signal metrics and tasks. We welcome other investigators to include this measure of spatial correlation in their research and, for this reason, the code linked in this article includes an automated pipeline to run on BIDS-formatted multimodal datasets.

A particularly fruitful application of our method is in the investigation of multimodal functional connectivity of resting-state activity when ECoG and fMRI recordings are acquired simultaneously (Hacker et al., 2017; He et al., 2008; Keller et al., 2013; Kucyi et al., 2018). Our study was limited by the fact that fMRI and ECoG data were acquired on different sessions. Multimodal simultaneous recordings, on the other hand, offer the opportunity to estimate the dynamic changes in the degree of phase amplitude coupling over time (Murta et al., 2017) and spatial coupling over time, by computing, with our method, the optimal kernel width over sliding windows. However, simultaneous recordings suffer from some limitations that would impact the acquisition of high-quality fMRI data or ECoG data, such as radiofrequency-induced heating and lower gradient switching strength to signal degradation in the neighborhood of the electrodes, which locally distorts the magnetic field (Murta et al., 2017). Despite these technical limitations, we propose that a dynamical cross-modal assessment of ECoG-fMRI activity patterns might shed light on the fluctuations over time in the degree of spatial correlation of functional connectivity.

More accessible are simultaneous multimodal recordings of fMRI and scalp electroencephalogram (EEG), which do not require recruitment from patient populations (Ullsperger and Debener, 2010). We envision that the method proposed here might perform well for this type of recordings, considering that EEG activity grouped in frequency bands maps precisely to local generators observed with fMRI (Scheeringa et al., 2016). The essential prerequisite is that the brain activity measured with EEG is first localized to the putative generators as accurately as possible, for example by employing a beamformer approach, which has successfully been used in combination with fMRI (Brookes et al., 2009, 2008).

### 4.4. Conclusions

This study represents an empirical estimation of optimal Gaussian kernel width to maximize the spatial correlation and spatial specificity between ECoG and fMRI acquisitions of the same task in the same participant. The reported kernel width can be interpreted as the most appropriate smoothing parameter for multimodal studies. Due to its consistency across participants, frequency bands and apparent resilience to artifacts, we suggest that the measure of concavity reflects an intrinsic feature of the generic point-spread function linking the fMRI activation pattern to the neuronal sources. Overall, the findings of this study provide a series of recommendations for future multimodal ECoG-fMRI studies, which have become more prevalent in the recent years.

## Supplementary Material

Piantoni_Supplementry

## Figures and Tables

**Table 1:** Summary of the ECoG results for all the participants who performed a motor task while implanted with an ECoG grid. For each participant, the table reports the total number of electrodes (excluding those containing artifacts), the percentage of electrodes that showed a significant increase or decrease in activity, the maximum and minimum z-score across all electrodes.

participant	# electrodes	% significant electrodes	maximum z-score	minimum z-score

P01	47	63.83%	20.388	−5.019
P02	54	44.44%	17.695	−8.650
P03	79	46.84%	35.021	−3.477
P04	94	31.91%	9.766	−2.894
P05	61	31.15%	4.677	−5.103
P06	55	30.91%	9.822	−4.045
P07	61	52.46%	24.615	−5.567
P08	55	41.82%	20.198	−2.566
P09	112	24.11%	7.166	−4.919
P10	55	49.09%	23.489	−4.432
P11	111	45.05%	18.296	−3.560
P12	56	48.21%	15.561	−5.655
P13	63	44.44%	11.466	−5.311
P14	47	55.32%	19.531	−5.200
P15	95	30.53%	24.922	−3.341
P16	102	19.61%	14.143	−4.009
P17	112	58.93%	10.924	−5.003
P18	120	20.00%	7.231	−6.352
P19	120	28.33%	29.157	−3.457

**Table 2:** Summary of the fMRI results for all the participants who performed a motor task with 3T fMRI. For each participant, the table reports the total number of voxels included in the analysis, the percentage of voxels that showed a significant increase or decrease in activity, the maximum and minimum z-score across all voxels.

participant	# included voxels	% significant voxels	maximum z-score	minimum z-score

P01	25492	3.31%	13.431	−7.249
P02	23015	10.39%	16.738	−8.086
P03	23417	24.77%	19.187	−11.902
P04	25972	15.69%	9.496	−8.715
P05	21009	27.53%	16.731	−12.293
P06	21254	22.57%	15.582	−12.086
P07	28299	19.04%	16.931	−12.328
P08	21787	2.07%	10.760	−3.338
P09	24084	17.53%	15.278	−9.430
P10	23345	7.81%	16.833	−12.066
P11	22171	9.59%	21.848	−9.120
P12	23652	11.20%	13.697	−7.153
P13	21679	29.80%	16.355	−11.645
P14	22123	3.28%	9.485	−5.772
P15	21184	5.72%	7.607	−7.494
P16	21684	3.41%	7.116	−7.767
P17	25100	7.04%	19.300	−8.913
P18	25109	6.50%	12.183	−7.330
P19	26278	4.57%	10.612	−7.615
