# Supplementary material for: Size of the spatial correlation between ECoG and fMRI activity
Source: Neuroimage. Author manuscript; Available in PMC 2023 Nov 6. (PMC10627020; doi:10.1016/j.neuroimage.2021.118459)
Supplement: Piantoni_Supplementry [file NIHMS1939187-supplement-Piantoni_Supplementry.docx]

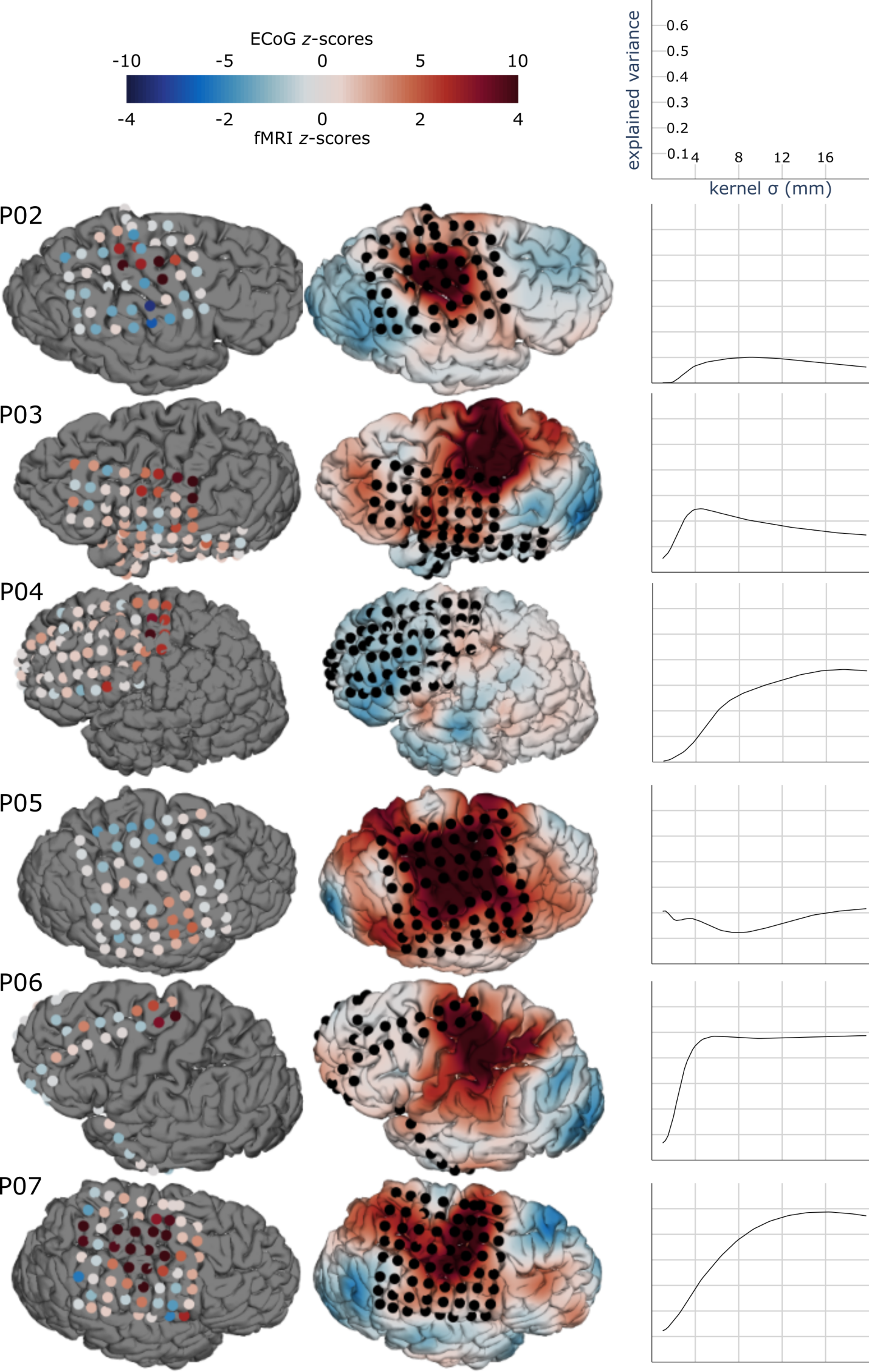


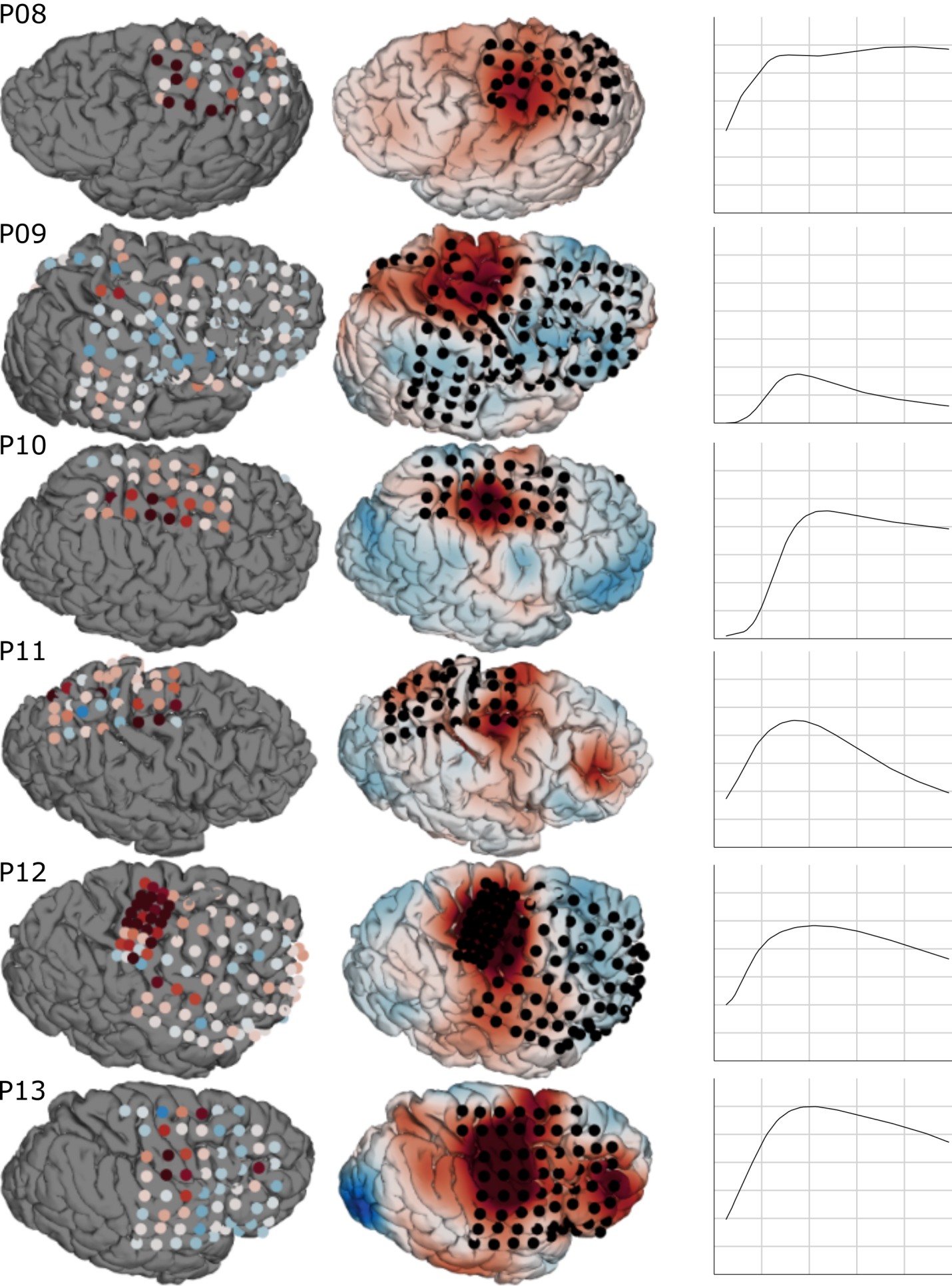


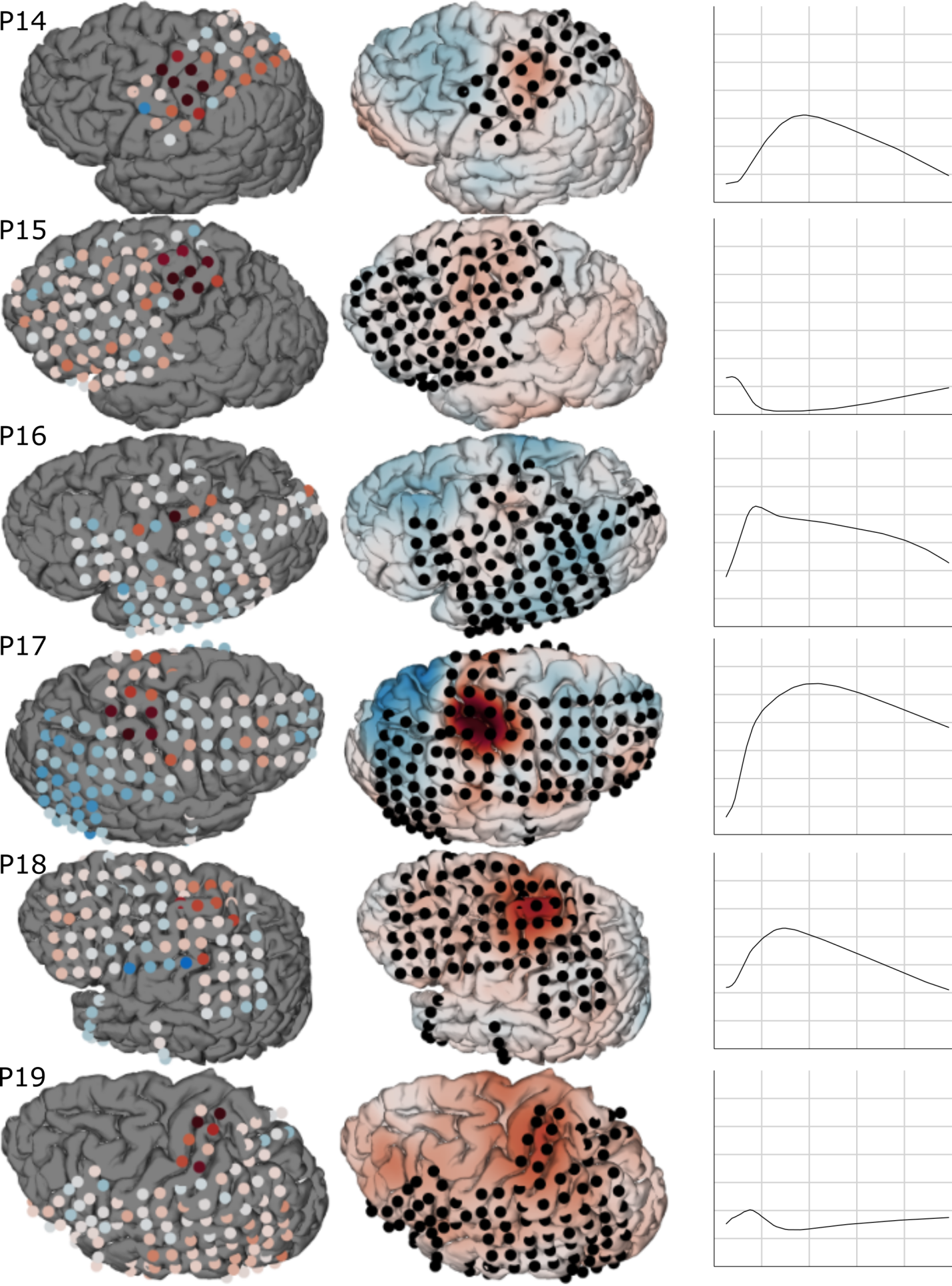


## Supplementary Figure S1, S2, S3

Spatial distribution of the z-scores for all the electrodes for all the participants (results for *P01* are shown in Fig. 2). Left-hand column shows the z-scores for the tasks performed while patients were implanted with ECoG electrodes. Middle column shows the z-scores for the tasks performed in the fMRI scanner. Because the z-scores for the ECoG tasks were overall higher than the z-scores for the fMRI tasks, the colorbar uses a different range for the two modalities. Right-hand column shows the correlation, measured as explained variance (r^2^), between fMRI and ECoG z-scores, as a function of the width of the Gaussian kernel.
